# A Chinese Patient with Spastic Paraplegia Type 4 with a *De Novo* Mutation in the SPAST Gene

**DOI:** 10.1155/2021/6636855

**Published:** 2021-12-14

**Authors:** Li Xu, Zijuan Peng, Chunhui Zhou, Jiqing Wang, Hunjin Luo, Qin Lu, Zhengjun Bao

**Affiliations:** ^**1**^ Antenatal Diagnosis Center of Women and Children Healthcare Hospital of ZHUZHOU, Zhouzhou, Hunan 412000, China; ^2^Central Hospital of ZHUZHOU, Affiliated Zhuzhou Hospital of Xiangya School of Medicine, Central South University, Zhouzhou, Hunan 412000, China; ^3^Women and Children Healthcare Hospital of ZHUZHOU, Zhouzhou, Hunan 412000, China; ^4^GeneTalks Biotech Co., Ltd., Changsha, Hunan 410000, China; ^5^Xiangnan University, Chenzhou, Hunan 423000, China

## Abstract

**Background:**

Spastic paraplegia type 4 (SPG4) is the most common type of hereditary spastic paraplegia (HSP) caused by mutations in the SPAST gene. *Case Presentation*. We report the case of a 27-year-old pregnant Chinese woman with HSP in whom we identified a missense mutation in the SPAST gene (c.1496G>A, p.Arg499His) and a nonsense mutation in the NEFH gene (c.289G>T, p.Glu97^*∗*^) via whole-exome sequencing; this finding corroborated that of Sanger sequencing. The patient exhibited the pure SPG4 phenotype with onset during childhood. The SPAST mutation was absent in the parents and paternal relatives. However, the NEFH mutation was identified in five people with no clinical phenotype. Based on theoretical conjecture and the family gene segregation information, we concluded that the SPAST mutation, but not the NEFH mutation, accounted for the proband's phenotype. Eventually, the woman gave birth to a healthy baby girl with the NEFH mutation.

**Conclusion:**

In this report, we identified a missense mutation in the SPAST gene (p.Arg499His) in a 27-year-old pregnant Chinese woman with HSP. We believe that this study expands the knowledge about the clinical parameters and mutation spectrum of SPG4.

## 1. Introduction

Hereditary spastic paraplegia (HSP), also known as Strümpell–Lorrain disease, is characterized by progressive muscle weakness and hypermyotonia in both lower extremities [1–4]. The main pathological changes observed in HSP are degeneration of the bilateral corticospinal tracts, with axonal degeneration and demyelination of the upper motor neurons in the long upper and lower fiber bundles (corticospinal and dorsal tracts) of the spinal cord [5]. HSP can be categorized into pure and complicated HSP depending on whether additional neurological or psychiatric symptoms are present [6]. HSP is caused by mutations in 72 different genes (SPG1–SPG72) [7]. Spastic paraplegia type 4 (SPG4) is the most common form of HSP, accounting for approximately 50% of the HSP genotypes [8, 9].

Charcot–Marie–Tooth (CMT) disease is the most common type of hereditary peripheral neuropathy. It is characterized by distal sensory loss, progressive distal muscle weakness, and atrophy [10, 11]. Genetic analysis is important for the differential diagnosis of HSP and CMT disease, which clinically overlap in terms of phenotype and pathogenesis. Here, we report a case of a *de novo* mutation in a 27-year-old pregnant woman with pure HSP associated with dyskinesia in both lower extremities. A *de novo* heterozygous mutation (NM_014946.4: c.1496G>A, p.Arg499His) in the SPAST gene and a heterozygous mutation (NM_021076.4: c.289G>T, p.Glu97^*∗*^) in the NEFH gene were found in the proband via whole-exome sequencing, which was then validated by Sanger sequencing. Furthermore, the mutation in the SPAST gene was identified as a causal variant. Eventually, the woman gave birth to a healthy baby girl with the NEFH mutation. This study may expand the clinical pathogenesis and variant spectrum of SPG4.

## 2. Case Presentation

**Figure 1:**
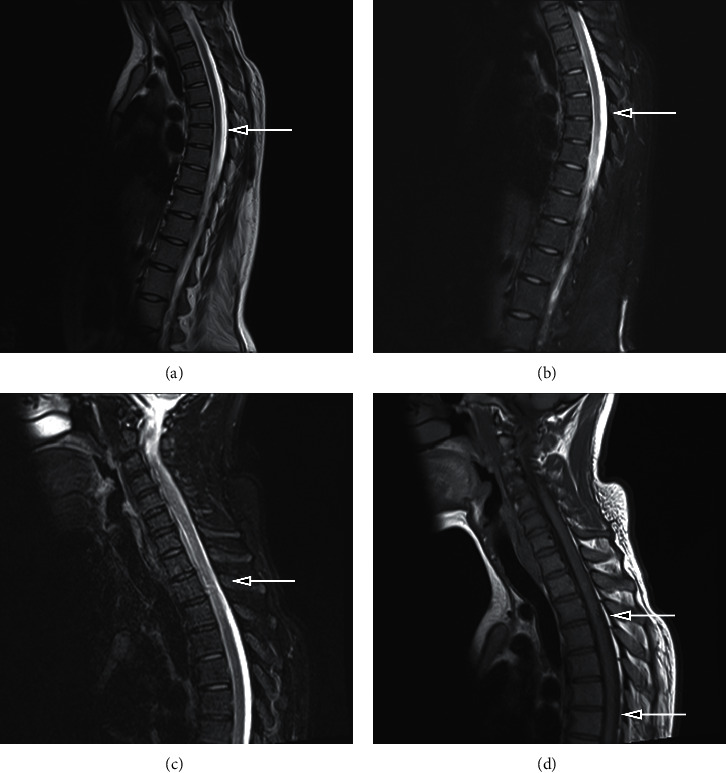
Magnetic resonance imaging (MRI) of the thoracic spinal cord. The gray arrow indicates the site of the thoracic spinal cord was smaller than the healthy people.

A 27-year-old woman who was pregnant (III-4) for more than 4 months was admitted to our hospital for genetic counseling and prenatal diagnosis. The woman had been born via vaginal delivery at full-term after an uneventful pregnancy. Her elder sister was healthy. She began to walk independently at 18 months of age. However, she developed progressive symptoms. At 11 years of age, she needed support to walk, and by 15 years of age, she was unable to stand or walk autonomously. At 22 years of age, she began to suffer from slow speech, slurred pronunciation, and occasional choking on food. Neurological examination revealed that the patient's proximal muscle strength in the upper limbs was V+/V, whereas that in the lower limbs was I/II. She was unable to straighten her knee, and the tendons in her lower extremity were hyper-reflexive, with a positive Babinski sign. Fortunately, she was intellectually normal and had no cerebellar, sensory, or autonomic dysfunctions. Furthermore, routine laboratory tests were normal. However, magnetic resonance imaging revealed thoracic spinal cord atrophy (Figure 1).

Genomic DNA was extracted from the peripheral blood of the proband, and single-nucleotide polymorphism array and karyotyping were normally performed and verified. Then, whole-exome sequencing was performed. First, the variants were filtered based on group frequency, and the variants with frequencies higher than 0.05 were excluded. Then, the phenotype was taken into account. Based on the output of Exomiser, the patient's standardized phenotypes (HPO) and associated gene phenotypes were matched and manually checked. Thereafter, the variants were classified according to the American Society of Medical Genetics and Genomics (ACMG) guidelines, and only those variants classified as above uncertain significance were included. Finally, only two candidate variants of the SPAST gene (c.1496G>A, p.Arg499His) and NEFH gene (c.289G>T, p.Glu97^*∗*^) were identified in the proband.

**Figure 2:**
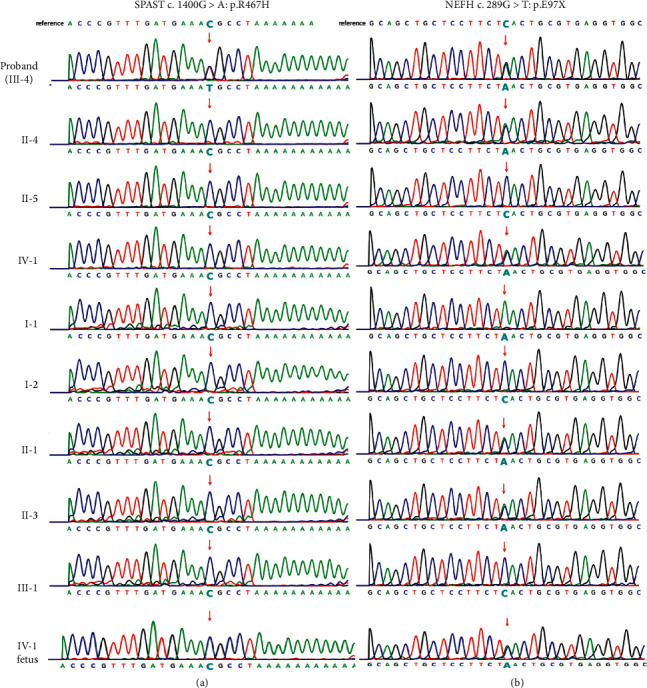
Sanger sequencing results of the SPAST and NEFH genes of the patient's family. (a) Mutation in the SPAST gene in a Chinese family. (b) Mutation in the NEFH gene in a Chinese family.

**Figure 3:**
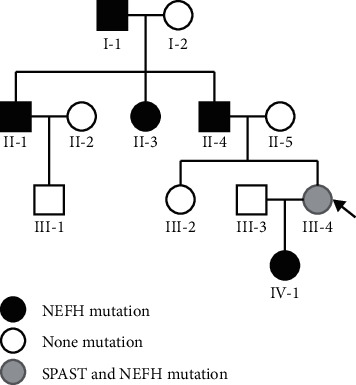
Pedigree of the patient's family. The gray arrow indicates the proband II:5 of the SPAST and NEFH mutations. Squares indicate males, and circles indicate females. Individuals carrying the NEFH mutation are represented by black-filled symbols, whereas empty symbols indicate healthy individuals without the NEFH or SPAST mutation. The presence of the SPAST mutation alone was not observed in any family member.

**Figure 4:**
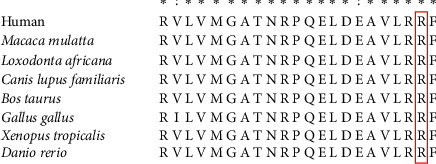
Sequence conservation analysis of the SPAST gene. Spastin protein sequence alignment across species showing the area of the amino acid substitution (red frame) and the surrounding residues.

We then investigated the presence of mutations in the SPAST and NEFH genes in the patient's paternal relatives via Sanger sequencing (Figure 2). This variant had the same amino acid changes as the known pathogenic variant, which was assessed as DM in the HGMD database (PS1). SPAST mutation is a *de novo* mutation (PM6, Figure 3). This SPAST mutation was not found in the East Asian Population Everyman Database (ExAC) (http://exac.hms.harvard.edu/) or Genome Aggregation Database (gnomAD) (http://gnomad.broadinstitute.org/) (PM2). The Mutation Taster (http://www.mutationtaster.org), Polyphen 2 (http://genetics.bwh.harvard.Edu/pph2/), and SIFT (http://provean.jcvi.org/genome_submit_2.php) software programs predicted that this SPAST variant as disease-causing, probably damaging, and deleterious, respectively (PP3). This mutation was located in a conservative site (Figure 4) and was present in the functional domain of the AAA + ATPase domain, which is essential for the function and activity of enzymes [12] and is a hotspot of pathogenic variants (PM1). Taken together, we identified the ACMG criteria for this mutation as PS1, PM1, PM2, PM6, and PP3, indicating that this variant is pathogenic.

Sanger sequencing reconfirmed the presence of the p.Glu97*∗* mutation in the NEFH gene, which was heterozygous in four paternal relatives. According to the ACMG guidelines [13], the c.289G>T mutation in the NEFH gene is a significant unknown mutation site that is associated with autosomal dominant diseases (CMT disease, axonal, type 2CC). Moreover, this variation was not found in ExAC. In gnomAD, the frequency of this variation was 1.81646*E* − 05; accordingly, PM2 was assigned.

Deletions in the C-terminal KSP repeat region of the NEFH gene in 5 of 356 patients with sporadic amyotrophic lateral sclerosis (ALS) and frameshift mutations (c.3008_3009del and c.3043_3044del) often lead to the translation of 40 additional amino acids encoding a cryptic amyloidogenic element, resulting in CMT disease [14, 15]. In contrast, termination of translation has occurred in the first exon of the NEFH gene (c.289G>T, p.Glu97*∗*), possibly resulting in nonsense-mediated mRNA decay. However, the additional cryptic amyloidogenic element (CAE) in the 3′-untranslated region (3′-UTR) is the key disease mechanism. Furthermore, sufficient evidence that these LOF gene variants result in CMT disease is not present. In the patient's family, the people with a truncated mutation in the NEFH gene were asymptomatic. Therefore, the PVS1 criteria were not applicable. We obtained the criteria PM2 and PP3 according to the ACMG guidelines and classified the mutation as uncertain significance.

Taken together, the variation in the SPAST gene (c.1496G>A, p.Arg499His) was the underlying cause of HSP in the pregnant women.

Because the patient was heterozygous for the variant, the probability that fetus had the variant was 0.5. Therefore, prenatal diagnosis was necessary to identify the zygosity of the variant in the fetus. After obtaining informed consent, amniocentesis was performed, and 30 ml of amniotic fluid was collected from the proband. DNA was extracted from the amniotic fluid using a genomic DNA extraction kit (Biochan). Then, Sanger sequencing was performed to determine whether the fetus carried the SPAST and NEFH gene mutations. The results revealed that the fetus had the NEFH mutation (c.289G>T, p.Glu97*∗*) but did not have the SPAST mutation (Figure 2, IV-1). Based on these results, the proband decided to continue the pregnancy. On September 23, 2019, she gave birth to a healthy baby girl weighing 2.35 kg. The baby is now growing up healthily.

## 3. Discussion

In the present study, we found a patient with mutations in the SPAST and NEFH genes. The patient had symmetrical weakness in the lower extremities, but the upper limbs were less affected; the progressive symptoms were childhood-onset. She presented with hypertonia and hyper-reflexia in her lower limb but with no obvious atrophy. Besides, she has a positive Babinski's sign. Based on these findings, we concluded that her upper motor neurons were mainly involved and that HSP was the most likely diagnosis. Furthermore, most of the phenotypes met the diagnostic criteria of “pure” HSP. However, she had difficulties in swallowing and speaking without cognitive impairment, indicating that the muscles of the pharynx or larynx may also be involved. Then, Sanger sequencing demonstrated that the SPAST mutation was only present in our patient but not in her relatives, whereas the NEFH mutation was present in our patient as well as in five parental-side relatives. However, none of the individuals with the NEFH mutation showed clinical manifestations. Based on the clinical phenotype, family information, and ACMG guidelines, we concluded that the missense mutation in the SPAST gene was the cause of her illness.

HSP, CMT, and ALS are autosomal dominant diseases with genetic heterogeneity and can lead to weakness in both lower extremities [8, 14, 16]. ALS is characterized by the death of motor neurons in the brain, brainstem, and spinal cord and involves the axons and myelin of both upper and lower motor neurons [16]. ALS is usually an adult-onset condition; the patient typically dies within 5 years of diagnosis. The affected individuals often present with either asymmetric focal weakness in the extremities (stumbling or poor handgrip) or bulbar findings (dysarthria or dysphagia) [17].

The clinical phenotype of HSP mainly involves the axons of upper motor neurons [18, 19], followed by the subsequent manifestation of leg spasticity, weakness, hypertonia, and hyper-reflexia. However, in some types of HSPs, both upper and lower motor neurons could be affected, with the manifestation of lower limb muscle atrophy [20]. In contrast, CMT disease primarily involves the axons and myelins of lower motor neurons and sensory nerves and predominantly affects the lower limbs, resulting in muscle weakness and atrophy. Furthermore, distal sensory impairment could be observed [14, 21, 22]. Therefore, genetic analysis is important for the differential diagnosis of HSP and CMT disease. In our study, mutations in the SPAST and NEFH genes were found in probands.

SPAST gene mutation is the main form of SPG4, which accounts for 50% of the HSP genotypes [23]. The SPAST gene is located on chromosome 2q22.3 and contains 17 coding exons. It encodes the protein spastin, which contains two structural domains: the microtubule interacting and trafficking domain and catalytic AAA domain (346–475) [24]. The AAA region catalyzes microtubule cutting, thereby playing a vital role in the transport of substances from the nerve cells [24]. More than 200 different mutations located at sites within the AAA region have been identified in patients with SPG4 [25]. As shown in Table 1, there were 19 patients in which p.Arg499His or p.Arg499Cys was casual variant for HSP with walking difficulties and lower limbs spasticity. It is onset at an early age and some with mental deficiency and dysarthria, except for p.Arg499His (4/6). In the present study, the patient also had walking and speech difficulties, Babinski sign, but absent in brain damage, contracture of the achilles tendon, and crab-like changes and intelligence delays. In addition, she emerged with thoracic spinal cord atrophy, which has never been found before.

Neurofilaments, which play an important role in cytoskeleton formation, contain three subunits: NEFL (light), NEFM (medium), and NEFH (heavy) [33]. Recent studies have suggested that the NEFH mutation is associated with the pathogenesis of sporadic ALS and CMT disease [15, 34]. And, the NEFH mutations associated with CMT disease often result in the loss of the stop codon as well as in frameshift mutation, eventually leading to the translation of an additional CAE in the 3′-UTR, which promotes abnormal protein aggregation but cannot form a network structure; this results in the inhibition of axonal growth and the eventual occurrence of CMT2-CC [14]. Consistent with these mechanisms, most of the reported mutation sites are close to terminators, resulting in delayed termination of protein translation (e.g., c.3010_3011delGA, c.3017_3020dup [34], c.3008_3009del, c.3043_3044del [14], and c.3057insG [35]). In our patient, the NEFH mutation site was located at the 289^th^ position, and protein translation was terminated 23 amino acids downstream, far from the 3′-UTR of the NEFH, thereby precluding CAE formation and not. The c.289G>T mutation site in the NEFH gene is already present in the Chinese population at a very low frequency. Previous studies and ClinGen dosage sensitivity evaluation (https://dosage.clinicalgenome.org/) indicate that the NEFH gene has no haploinsufficiency effect. Additionally, the heterozygous variant was observed in asymptomatic individuals. Therefore, we conclude that the heterozygous NEFH mutation is unrelated to the patient's phenotype.

Most of all, Sanger sequencing of the amniotic fluid revealed that the fetus had the NEFH mutation in our study. However, based on theoretical conjecture and genetic and phenotypic studies, we concluded that this fetus would be healthy and not exhibit the CMT2-CC-associated phenotype. Therefore, in the end, the woman chose to continue her pregnancy and gave birth to a healthy baby girl.

In conclusion, we encountered a case of childhood-onset pure SPG4 phenotype caused by a *de novo* mutation in the SPAST gene in a Chinese patient. Through prenatal diagnosis, we guided the woman to give birth to a healthy baby girl. This study may provide a clinical basis for further research and expand our knowledge about the variant spectrum of SPG4.

## Figures and Tables

**Table 1:** Clinical findings of hereditary spastic paraplegia.

Patient	Nucleotide change	Amino acid change	Sex	Duration age	Age of onset	Walking achievement	Functional impairment	Spasticity	Increased reflexes	Dysarthria	MRI or intelligence	Literature
1	c.1496G>A	p.Arg499His	M	12 y	14 m	No	6	3	Exaggerated muscle stretch reflexes of both the upper and lower limbs	9 y, lost ability to speak at 12 y	More pronounced hyperintense bilateral PLICs in FLAIR sequences	[26]
2	c.1496G>A	p.Arg499His	F	36 y	<2 y	NA	6	3	NA	<6 y	Mild hyperintensities in corticospinal tracts (white arrows) in T2-weighted and FLAIR sequences	[27]
3	c.1496G>A	p.Arg499His	F	13 y	20 m	No	5	LL spasticity	Lower limb hyper-reflexia	<11 y	Intellectual disability	[28]
4	c.1496G>A	p.Arg499His	F	5 y	1 y	22 m, hold on to furniture	4	NA	Yes	Early expressive language delays	Low-lying conus medullaris with minimal thickening of the filum terminale	[28]
5	c.1496G>A	p.Arg499His	M	11 y	1.5 y	48 m, walk alone	2	NA		No	NA	[29]
6	c.1496G>A	p.Arg499His	M	3 y	1 y	NA	NA	Spasticity	Lower extremity hyper-reflexia, Babinski sign	No	NA	[30]
7	c.1495C>T	p.Arg499Cys	M	20	13 y	NA	3	1	LL increased; UL normal/yes	No	NA	[31]
8	c.1496G>A	p.Arg499His	M	>40	Childhood	Limited walking without aid	3	3	Not determined	NA	NA	[31]
9	c.1495C>T	p.Arg499Cys	F	60	Childhood	NA	6	3	Lower limb increased; upper limb normal	NA	NA	[31]
10	c.1495C>T	p.Arg499Cys	NA	63	Childhood	Need help for daily life	6	3	LL, UL/bilateral	NA	NA	[32]
11	c.1495C>T	p.Arg499Cys	NA	55	51	Need help for daily life	2	1	LL, UL/bilateral	NA	NA	[32]
12	c.1495C>T	p.Arg499Cys	NA	53	Adolescence	Partially need help for daily life	5	3	LL, UL/bilateral	NA	Cortical and subcortial atrophy, nonspecific WMH	[32]
13	c.1495C>T	p.Arg499Cys	NA	47	4	Need help for daily life	6	3	LL, UL/bilateral	NA	Cortical atrophy	[32]
14	c.1495C>T	p.Arg499Cys	NA	45	5	None	5	3	LL, UL/bilateral	NA	NA	[32]
15	c.1495C>T	p.Arg499Cys	NA	43	Birth	Need help for daily life	6	3	LL/bilateral	NA	Cortical and subcortical atrophy	[32]
16	c.1495C>T	p.Arg499Cys	NA	39	Childhood	Partially need help for daily life	6	3	LL, UL/bilateral	NA	NA	[32]
17	c.1495C>T	p.Arg499Cys	NA	29	Birth	Partially need help for daily life	3	3	LL, UL/bilateral	NA	Normal	[32]
18	c.1495C>T	p.Arg499Cys	NA	27	1	Partially need help for daily life	3	2	LL/bilateral	NA	NA	[32]
19	c.1495C>T	p.Arg499Cys	NA	24	Childhood	Need help for daily life	3	2	LL/bilateral	NA	NA	[32]
20	c.1496G>A	p.Arg499His	F	27	Childhood	No	6	Yes	Lower limb increased, positive Babinski sign	Speech is slow, slurred, and the voice diminishes at 22 y	Thoracic spinal cord atrophy	This report

LL, lower limb; UL, upper limb. Functional impairment: 0—none, 1—no functional impairment but signs at examination, 2—mild, 3—moderate, 4—walking with one cane, 5—walking with two canes, and 6—wheelchair-bounded. Gait spasticity: 0—none, 1—mild, 2—moderate, and 3—severe.

## Data Availability

All data included in this study are available upon request from the corresponding author.

## References

[1] Depienne C., Stevanin G., Brice A., Durr A. (2007). Hereditary spastic paraplegias: an update. *Current Opinion in Neurology*.

[2] Chamard L., Ferreira S., Pijoff A., Silvestre M., Berger E., Magnin E. (2016). Cognitive impairment involving social cognition in SPG4 hereditary spastic paraplegia. *Behavioural Neurology*.

[3] Walusinski O. (2020). A historical approach to hereditary spastic paraplegia. *Revue Neurologique*.

[4] Nirmal A. K. (2020). A case study of hereditary spastic paraplegia. *The Journal of the Association of Physicians of India*.

[5] Tisher A., Salardini A. (2016). A case report of a woman with young onset cognitive impairment associated with hereditary spastic paraplegia due to a mutation in the SPAST gene. *Journal of the Neurological Sciences*.

[6] Kara E., Tucci A., Manzoni C. (2016). Genetic and phenotypic characterization of complex hereditary spastic paraplegia. *Brain*.

[7] Harding A. E. (1983). Classification of the hereditary ataxias and paraplegias. *The Lancet*.

[8] Yang J., Seo J. Y., Lee K.-W., Park H.-M. (2019). Novel pathogenic variant of SPAST (c.1413+4A>G) in a patient with hereditary spastic paraplegia. *Journal of Clinical Neurology*.

[9] Cui F., Sun L., Qiao J. (2020). Genetic mutation analysis of hereditary spastic paraplegia: a retrospective study. *Medicine (Baltimore)*.

[10] Azevedo H., Pupe C., Pereira R., Nascimento O. J. M. (2018). Pain in charcot-marie-tooth disease: an update. *Arquivos de Neuro-Psiquiatria*.

[11] Akdal G., Kocoglu K., Tanriverdizade T. (2020). Vestibular impairment in charcot-marie-tooth disease. *Journal of Neurology, Neurosurgery, and Psychiatry*.

[12] Depienne C., Fedirko E., Faucheux J.-M. (2007). A de novo SPAST mutation leading to somatic mosaicism is associated with a later age at onset in HSP. *Neurogenetics*.

[13] Richards S., Aziz N., Aziz N. (2015). Standards and guidelines for the interpretation of sequence variants: a joint consensus recommendation of the American college of medical genetics and genomics and the association for molecular pathology. *Genetics in Medicine*.

[14] Jacquier A., Delorme C., Belotti E. (2017). Cryptic amyloidogenic elements in mutant NEFH causing Charcot-Marie-Tooth 2 trigger aggresome formation and neuronal death. *Acta Neuropathologica Communications*.

[15] Rooke K., Figlewicz D. A., Han F.-y., Rouleau G. A. (1996). Analysis of the KSP repeat of the neurofilament heavy subunit in familial amyotrophic lateral sclerosis. *Neurology*.

[16] Mitchell J. D. (2000). Guidelines in motor neurone disease (MND)/amyotrophic lateral sclerosis (ALS)–from diagnosis to patient care. *Journal of Neurology*.

[17] Kunst C. B. (2004). Complex genetics of amyotrophic lateral sclerosis. *The American Journal of Human Genetics*.

[18] Yu W., Jin H., Deng J., Nan D., Huang Y. (2020). A novel SPAST gene mutation identified in a Chinese family with hereditary spastic paraplegia. *BMC Medical Genetics*.

[19] Warner T. T. (2020). Clinical and pathogenic themes in hereditary spastic paraplegia. *Brain*.

[20] Parodi L., Fenu S., Stevanin G., Durr A. (2017). Hereditary spastic paraplegia: more than an upper motor neuron disease. *Revue Neurologique*.

[21] Rossor A. M. (2020). Lessons from late onset charcot‐marie‐tooth disease. *Journal of the Peripheral Nervous System*.

[22] Nagappa M., Sharma S., Taly A. B. (2020). Charcot marie tooth. *StatPearls*.

[23] McDermott C. J., Burness C. E., Kirby J. (2006). Clinical features of hereditary spastic paraplegia due to spastin mutation. *Neurology*.

[24] Murphy S., Gorman G., Beetz C. (2009). Dementia in SPG4 hereditary spastic paraplegia: clinical, genetic, and neuropathologic evidence. *Neurology*.

[25] Shoukier M., Neesen J., Sauter S. M. (2009). Expansion of mutation spectrum, determination of mutation cluster regions and predictive structural classification of SPAST mutations in hereditary spastic paraplegia. *European Journal of Human Genetics*.

[26] Ogasawara M., Saito T., Koshimizu E., Akasaka N., Sasaki M. (2019). A p.Arg499His mutation in SPAST is associated with infantile onset ascending spastic paralysis complicated with dysarthria and anarthria. *Neuropediatrics*.

[27] de Souza P. V. S., Bortholin T., Naylor F. G. M., de Rezende Pinto W. B. V., Oliveira A. S. B. (2016). Infantile-onset ascending spastic paraplegia phenotype associated with SPAST mutation. *Journal of the Neurological Sciences*.

[28] Gillespie M. K., Humphreys P., McMillan H. J., Boycott K. M. (2018). Association of early-onset spasticity and risk for cognitive impairment with mutations at amino acid 499 in SPAST. *Journal of Child Neurology*.

[29] Polymeris A. A., Tessa A., Anagnostopoulou K. (2016). A series of Greek children with pure hereditary spastic paraplegia: clinical features and genetic findings. *Journal of Neurology*.

[30] Park S.-Y., Ki C.-S., Kim H.-J. (2005). Mutation analysis of SPG4 and SPG3A genes and its implication in molecular diagnosis of Korean patients with hereditary spastic paraplegia. *Archives of Neurology*.

[31] Depienne C., Tallaksen C., Lephay J. Y. (2006). Spastin mutations are frequent in sporadic spastic paraparesis and their spectrum is different from that observed in familial cases. *Journal of Medical Genetics*.

[32] Ribaï P., Depienne C., Fedirko E. (2008). Mental deficiency in three families with SPG4 spastic paraplegia. *European Journal of Human Genetics*.

[33] Lee M., Xu Z., Wong P., Cleveland D. (1993). Neurofilaments are obligate heteropolymers in vivo. *Journal of Cell Biology*.

[34] Rebelo A. P., Abrams A. J., Cottenie E. (2016). Cryptic amyloidogenic elements in the 3′ UTRs of neurofilament genes trigger axonal neuropathy. *The American Journal of Human Genetics*.

[35] Bian X., Lin P., Li J. (2018). Whole-genome linkage analysis with whole-exome sequencing identifies a novel frameshift variant in NEFH in a Chinese family with charcot-marie-tooth 2: a novel variant in NEFH for charcot-marie-tooth 2. *Neurodegenerative Diseases*.

